# Historical Accountability for Equitable, Efficient, and Sustainable Allocation of the Right to Emit Wastewater in China

**DOI:** 10.3390/e20120950

**Published:** 2018-12-10

**Authors:** Jin Huang, Van Butsic, Weijun He, Dagmawi Mulugeta Degefu, Zaiyi Liao, Min An

**Affiliations:** 1 College of Economics & Management, Three Gorges University, Yichang 443002, China; 2 Department of Environmental Science, Policy and Management. University of California Berkeley, Berkeley, CA 94720-3114, USA; 3 Faculty of Engineering and Architectural Science, Ryerson University, Toronto, ON M5B 2K3, Canada; 4 Business School, Hohai University, Nanjing 211100, China

**Keywords:** wastewater discharge permits, province deviation coefficient, information entropy, historical responsibility, national-provincial level

## Abstract

Establishing policies for controlling water pollution through discharge permits creates the basis for emission permit trading. Allocating wastewater discharge permits is a prerequisite to initiating the market. Past research has focused on designing schemes to allocate discharge permits efficiently, but these schemes have ignored differences among regions in terms of emission history. This is unfortunate, as fairness may dictate that areas that have been allowed to pollute in the past will receive fewer permits in the future. Furthermore, the spatial scales of previously proposed schemes are not practical. In this article, we proposed an information entropy improved proportional allocation method, which considers differences in GDP, population, water resources, and emission history at province spatial resolution as a new way to allocate waste water emission permits. The allocation of chemical oxygen demand (COD) among 30 provinces in China is used to illustrate the proposed discharge permit distribution mechanism. In addition, we compared the pollution distribution permits obtained from the proposed allocation scheme with allocation techniques that do not consider historical pollution and with the already established country plan. Our results showed that taking into account emission history as a factor when allocating wastewater discharge permits results in a fair distribution of economic benefits.

## 1. Introduction

Total pollution load regulation controls total wastewater emission and can be used to control environmental quality [1]. Such policies have been applied in developed countries such as United States of America and Japan [2,3,4,5]. Developing countries like China are also experimenting with similar regulation. These schemes make the discharge permits a scarce resource. As a result, different regions compete for allocation permits. Each region desires to gain higher wastewater emission permits, since emission permits directly influences economic development [6,7]. Therefore, the uneven distribution of wastewater discharge permits leads to the uneven distribution of economic benefits [8,9,10,11]. 

Careful design of wastewater policy is necessary for these policies to be effective [12,13]. One factor that needs to be considered to achieve this goal is to align the spatial resolution of the allocation policy and the political management zones. Most of the previous studies in China were done at the river basin spatial scale and mainly focused on quantifying the impact of the upstream activities on the downstream allocation [14,15,16,17]. Studies at practical spatial management scales are very important for the implementation of proposed emission allocation schemes. This is the reason why our study is conducted at the province scale. 

In addition to the spatial scale, any allocation framework should take in to account the historical emission trend of the provinces in order to ensure fair distribution of discharge permits [18,19]. Considering historical responsibility while allocating permits has been extensively used in greenhouse-gas emission quota allocation [20,21]. Wastewater discharge permits are often allocated through auctioning or grandfathering. Auctions aim to create economically efficient distributions and have been widely used in carbon markets [22,23]. However, due to the huge potential cost of non-cooperation among stakeholders, the distribution mechanism of auctioning has been increasingly questioned [24]. One of the big challenges in wastewater emission permit allocation is fitting historical responsibility into the existing allocation frameworks. Grandfathering takes historical responsibility into account but is not efficient. 

Aiming to mitigate the above drawbacks, we proposed a new wastewater permit distribution mechanism that takes into account fairness by considering emission history at the provincial scale. As one of the main wastewater discharge pollutants, the allocation of chemical oxygen demand (COD) emission permits among the 30 provinces of China is used to illustrate the proposed mechanism and compare results with the existing country plan as well as with the results from methods proposed by recent studies.

## 2. Methods

### 2.1. Study Area

**Figure 1:**
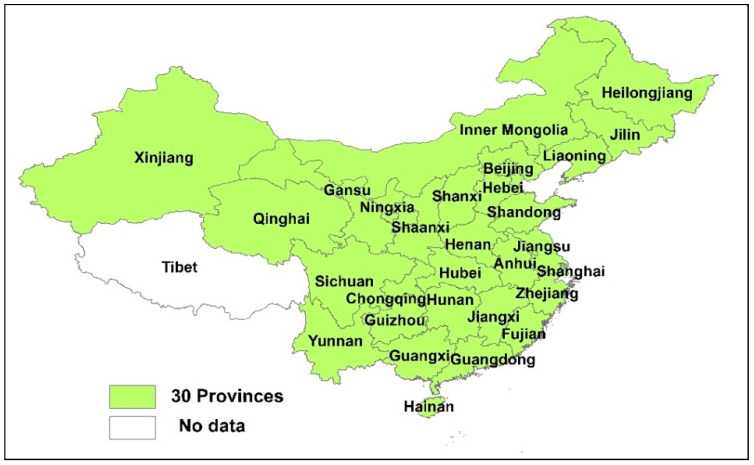
The 30 provinces of mainland China.

The objective of this research is to distribute China’s 2020 wastewater COD discharge permits among 30 provinces (As shown in Figure 1, Tibet is not included because it has little wastewater discharge and the historical data is also unavailable) with 2015 as the target year for the allocation. Since 1996, the Chinese government has developed a total pollution load control policy and allocated wastewater discharge permits to each administrative province every five years based on current pollution load [7]. However, the central government’s uniform pollution permit distribution scheme does not consider each province’s social, economic, and environmental differences. Hence, the allocation may not respect the principle of equity and could cause big dissatisfaction among the provinces [6]. With increasing water pollution in China [25], wastewater discharge allocation policy that is fair to all provinces is very important for sustainable development in China.

China’s water resource management structure is based on political management structure rather than on geographic location [14]. Usually, the first step is for the central government’s Environmental Protection Bureau to decide the total wastewater discharge permit for each province [6,7]. This step is very crucial and challenging because each province is autonomous and competes for more emission discharge permits since a higher discharge permit can be translated directly to more economic benefits. As a result, each province acts as a utility-maximizing agent.

Mainland China’s 30 provinces differ greatly in their economic growth, social features, and environment [26]. Previously, to promote economic prosperity, the Chinese government allowed some provinces to pollute more without bearing the cost of pollution [27]. Currently, the Chinese government is attempting an environmentally friendly development path and is internalizing pollution cost within development efforts. Applying such policy equally to all the 30 provinces might not be fair because these provinces have different socio-economic and environmental makeups but most importantly their emission history is highly asymmetric. 

### 2.2. Data

The period for the study is from 2000 to 2020. This period covers two decades of major economic growth for China and is a period with well-documented data. The gross domestic product (GDP) and population data of each province for the period from 2000–2015 are obtained from China Statistical Yearbook [28]. Each province’s chemical oxygen demand (COD) emission data from for the study period is obtained from China Environment Yearbook [29]. The water capital of each province for the time period under consideration were extracted from China Water Resources Bulletin [30]. To eliminate the impact of inflation, the GDP was deflated by using 2000 as the base year.

### 2.3. Historical Pollution and Emissions Discrepancies

Before distributing the total wastewater discharge permits, we need to understand the differences among provinces in terms of pollution history to ensure equity. In this part, we choose GDP as one factor, to analyze the historical discrepancies among provinces. 

Assume n is the number of provinces in China and *m* represents the *m*th province. 

For the period from year *x* to *y*, $gdp_{xy}^{m}$ is the *m*th province’s GDP proportion and can be determined as follows:

(1) $$gdp_{xy}^{m}=\frac{{\mathrm{GDP}}_{xy}^{m}}{\sum_{n=1}^{30}{\mathrm{GDP}}_{xy}^{n}}$$

For the period *x* to *y*, $cod_{xy}^{m}$ is the *m*th province’s COD proportion and can be computed as follows:

(2) $$cod_{xy}^{m}=\frac{{\mathrm{COD}}_{xy}^{m}}{\sum_{n=1}^{30}{\mathrm{COD}}_{xy}^{n}}$$

Then, province deviation coefficient can be calculated as:

(3) $$\varepsilon_{\mathrm{GDP}}=\frac{cod_{xy}^{m}}{gdp_{xy}^{m}}$$

If $\varepsilon_{\mathrm{GDP}}$ is greater than one, this indicates that the proportion of historical COD emission in this province is greater than the proportion of GDP. This means the province’s GDP increase is associated with decreasing quality of the aquatic environment as the result of increased COD emissions. Using the same procedure, we can obtain $\varepsilon_{population}$ and $\varepsilon_{water\;amount}$. If the values of deviation coefficients for these parameters are greater than 1, then COD emission increases proportionally with GDP, population, and water capital. These three dimensions provide insight on whether each province’s development path needs to change. For example, when a province’s $\varepsilon_{\mathrm{GDP}}>1$, it indicates that the environment is disproportionately damaged and the development path should change to a more environmentally friendly economic development model.

### 2.4. Entropy-Improved Proportional Method for Wastewater Discharge Permit Allocation

The entropy method is an objective way of weighing and measuring the disorder of a system [31,32]. By calculating entropy, we determine the weight that reflects the different pollution level among provinces [33].

The disadvantage of this method is that it does not consider the differences among the provinces. Using the entropy method avoids this shortcoming. Hence the wastewater emission permit allocation satisfies the principles of equity, efficiency, and sustainability. The detailed procedure is as follows.

Suppose the status of province *i*’s emission is *q_i_*, *a* is the GDP index, population, and water capital.

$x_{ia}$ is the value of province i from index *a*.

The first step is the standardization of $x_{ia}$:

For the positive index: $x_{ia}^{\prime }=\frac{x_{ia}-\min x_{a}}{\max x_{a}-\min x_{a}}$.

For the negative index: $x_{ia}^{\prime }=\frac{\max x_{a}-x_{ia}}{\max x_{a}-\min x_{a}}$.

Here, we considered population and water capital as indicators. There are some principles for reducing wastewater discharge permits. First, those with higher GDP should reduce more because they have greater ability and resources to reduce COD emission while maintaining their economic development. Second, provinces with higher population should cut less since every person has an equal right to COD emission. Third, those with more water capital should cut down less because they can assimilate more COD. Thus, we can see that GDP is a positive index, while population and water capital are negative indices.

The weight $b_{ia}$ of province i from index *a* can be determined as follows.

(4) $$b_{ia}=\frac{x_{ia}^{\prime }}{\sum_{n=1}^{m}x_{ia}^{\prime }}$$

The information entropy ($e_{a}$) of province i from can index *a* be computed as follows.

(5) $$e_{a}=-\frac{1}{\ln n}\sum_{i=1}^{n}b_{ia}\times \ln b_{ia}$$

The weight of the index *a* is calculated using the entropy method [34,35].

(6) $$w_{a}=\frac{1-e_{a}}{\sum_{a=1}^{3}\left(1-e_{a}\right)}$$

The method we suggest here is the information entropy improved proportional allocation method. The equal allocation method reduces each province’s emission proportionally. Information entropy improved means that, according to the principle of fairness, provinces adjust the equal-proportional allocation method. The greater the differences among provinces, the greater the disparities in terms of emission reductions. On the contrary, when regional differences are negligible, the emission reductions of the provinces are more similar. 

Assuming the total target reduction rate is *b*, *q_i_* is the province *i*th COD emission for the base year (2015), the total COD emission of n provinces (n=30) participating in waste water discharge permit allocation for the base year (the year of 2015) is $q=\sum_{i=1}^{n}q_{i}$. 

Then, province *i*’s reduction rate $C_{i}$ will be:

(7) $$C_{i}=\bar{C}\times D_{i}^{\prime }$$

where, $\bar{C}$ is the average reduction rate for each province,$D_{i}^{\prime }$ is the relative difference of province *i*.

$D_{i}^{\prime }$ is determined by GDP, population and water capital indices. Firstly, each province’s difference score $D_{i}$ is calculated as,

(8) $$D_{i}=\sum_{a=1}^{3}x_{ia}^{\prime }\times w_{a}$$

Then, $D_{i}^{\prime }$ is computed as follows;

(9) $$D_{i}^{\prime }=\frac{D_{i}}{\frac{1}{n}\sum_{i=1}^{n}D_{i}}$$

The average reduction the rate $\bar{C}$ for province *i* will be:

(10) $$\bar{C}=\frac{b\times \sum_{i=1}^{n}q_{i}}{\sum_{i=1}^{n}\left(D_{i}^{\prime }\times q_{i}\right)}$$

Finally, the emission reduction and amount variables are determined as follows:

*i*th province’s emission reduction *R_i_*:

(11) $$R_{i}=C_{i}\times q_{i}$$

*i*th province’s emission amount *E_i_*:

(12) $$E_{i}=q_{i}-R_{i}$$

## 3. Results and Discussion

### 3.1. The Proposed Method’s Allocation Result and Its Internal Mechanism

The total wastewater discharge permits of each province based on the information entropy improved proportional allocation method are shown in Table 1. From Table 1, we can see that each province’s reduction rate is between 1% and 20%, the outputs from the proposed allocation method are within each province’s acceptable range [6,7,14]. Among all provinces, Jiangsu has the highest reduction rate of 15.5%, while Sichuan is the lowest at 5.58%. Shandong has the largest reduction amount and Qinghai has the smallest.

**Figure 2:**
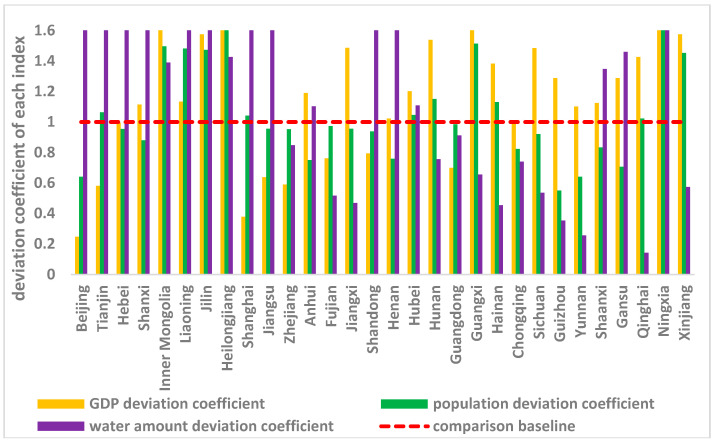
Province deviation coefficient of each index.

Economic development (GDP), population, and water capital are the main factors that influence wastewater discharge allocation [6,7,14]. Using historical data and the method we proposed, each index’s deviation coefficient was calculated, and the results are shown in Figure 2 (the detailed procedures are included in Appendix Table A1, Table A2 and Table A3).

From Figure 2 we can see that during the period from 2000 to 2015, only Zhejiang, Fujian, Guangdong, and Chongqing had COD emissions levels that correspond with low values of the three deviation coefficients. All three deviation coefficients were larger for Inner Mongolia, Liaoning, Jilin, Heilongjiang, Hubei, and Ningxia.

**Figure 3:**
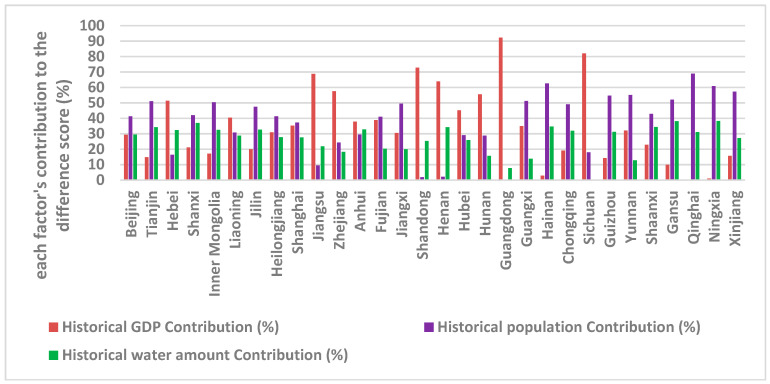
The contribution of GDP, population, and water capital to the allocation score.

Each province’s difference score (D_i_) shown in Table 1 indicates heterogeneity among the provinces. It not only includes the historical responsibility each province should bear, but also contains the information on GDP, population, and water capital difference among the provinces. In order to calculate this, the three indices’ historical features need to be combined into one comprehensive value for decision making. The weight assigned by the information entropy method to GDP, population, and water capital are 0.55, 0.28, and 0.17, respectively. With each province’s historical data of these three factors, each factor’s contribution to the wastewater discharge permit allocation difference score was calculated and the spatial trend is shown in Figure 3 (detailed results are included in the Appendix Table A4). 

In our proposed allocation method, Hebei, Liaoning, Jiangsu, Zhejiang, Anhui, Shandong, Henan, Hubei, Hunan, Guangdong, and Sichuan provinces’ allocations are mainly influenced by their GDP history. The other 19 provinces’ allocations are highly impacted by their population growth. The water capital of each province did not impact the allocation of the permits significantly for all provinces. In addition, it is important to keep in mind that each index’s weight could change if the study period is extended. Hence, the proposed allocation framework is flexible and the allocations from it can be adjusted through time. 

### 3.2. Comparison of Results with Those from Alternative Methods

**Figure 4:**
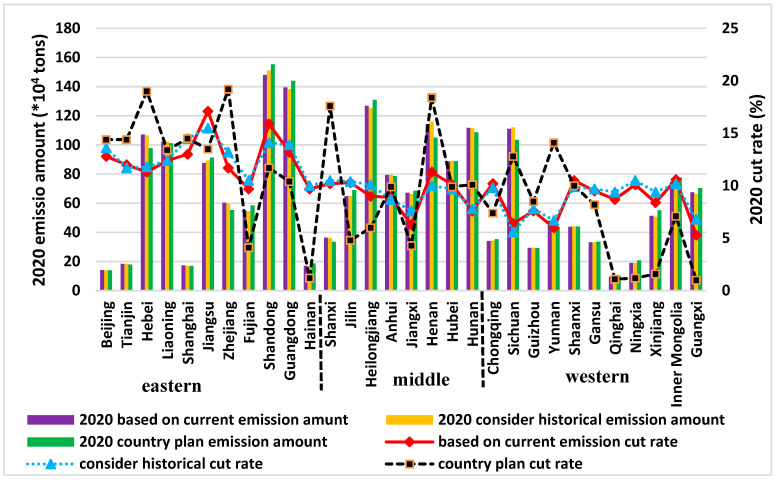
Chemical oxygen demand (COD) discharge permits and emission reduction rate for the target year 2020 from different allocation plans.

The allocation method integrated with emission history proposed in this paper contains the following two main pieces of information: (1) Differences among provinces in terms of GDP population number and water capital; (2) Disparities among the provinces in terms of historical emission responsibilities. By comparing the results with those obtained from alternative methods and the central government’s allocation plan, we can get a deeper understanding of the differences in the disparities between the results of the different allocation methods. The comparative results are shown in Figure 4 and each province’s emission amount and reduction rate can be found in Appendix Table A5.

**Figure 5:**
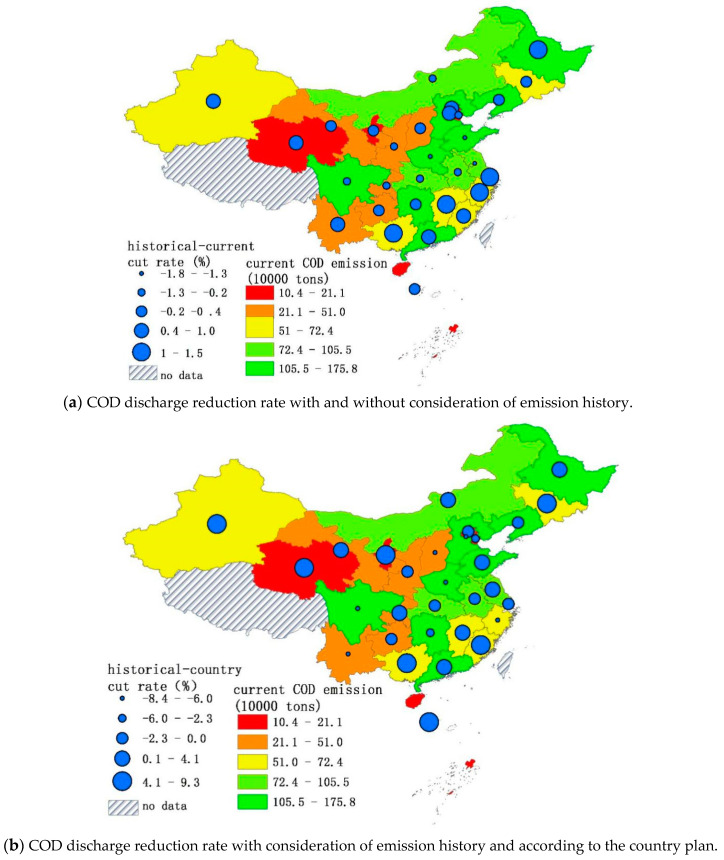
The difference in COD cut-rate with different methods. Map generated with ArcGIS 10.6 for desktop (http://www.esri.com/sofware/arcgis).

From Figure 4, we find that the emission cut rates’ lines are gentler for the methods based on current emissions. The eastern part of China’s emission cut-rate is relatively higher than the middle and western part of the country for the method based on current emission trends and for the proposed one. The country plan methods give excessively small reduction rates for the western part of China. Provinces like Qinghai, Ningxia, and Xinjiang, which are the sources of China’s major rivers, benefit from this trend. The differences among the different allocation plans in terms of emission amount are negligible. Figure 5a shows the proposed mechanism difference among the allocation outputs in terms of the reduction rate. If the results from the allocation that considers emission history is higher than the one that does not, it indicates that these provinces had already historically enjoyed lower COD emission costs. The lower reduction rate results from the distribution mechanism that takes the emission history of the provinces into account mean that these provinces need higher pollution permits to take their economy a step forward. Provinces with bigger emission reduction rates are mostly in southern China, or in water-scarce regions of China like Beijing city, Tianjin city, and Hebei Province.

The difference between the reduction rates from the allocation scheme that considers emission history and the one that does not is presented in Figure 5b. The Chinese government’s reduction plan usually considers each province’s economy and uses a uniform reduction rate [6,7]. Figure 5b shows that the reduction rate from our proposed method differs from the country plan. Let’s take Jilin and Zhejiang province in northeastern China as an example. As an industrial base for the time period 2000–2015, Jilin has a COD emission percentage increase of approximately 4.9% to 5.3% every year. Compared to Zhejiang’ reduction rate, the country plan allocates a smaller reduction rate to Jilin. 

**Figure 6:**
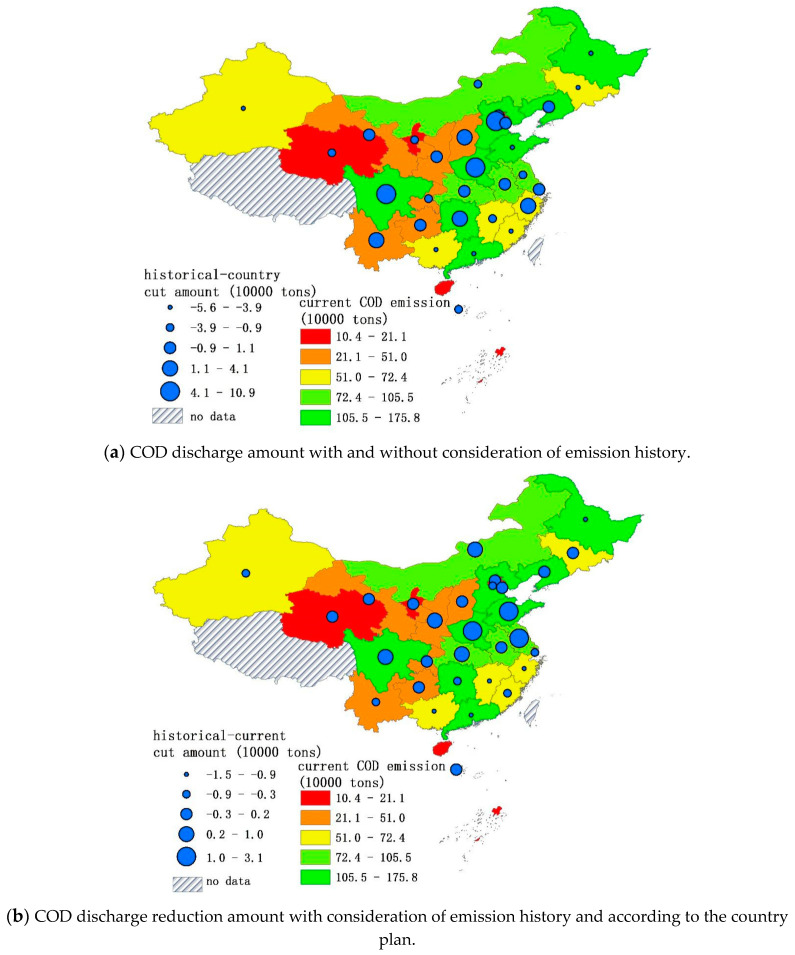
The difference in COD cuts with different methods. Map generated with ArcGIS 10.6 for desktop (http://www.esri.com/sofware/arcgis).

Figure 6a shows the difference in terms of emission amount from allocation schemes that do and do not take the emission history of the provinces and the country plan. By comparing the reduction rate of the current and historical scenarios, the government can approximate the historical responsibility of each region and determine whether each region’s rate of discharge should decrease or not. We took Guangdong, Guangxi, Ningxia, and Jiangsu as representative provinces for deeper analysis because these provinces’ allocations from these schemes differ greatly. Guangdong is a developed province and Guangxi is an underdeveloped province. According to the “Sustainable Cities in China”, 2016 report, these two provinces’ capital cities have severe water pollution problems which are affecting their sustainable development. However, under the country plan, both them do not have high COD reduction rates. 

Ningxia, in the western part of China, is one of the most water-scarce provinces [36]. The population of this province is low, therefore the discharge quota should be low and the reduction rate should be higher because the region does not have large assimilation capacity. However, the country plan only considers the GDP and assigns a very low reduction rate to this region. On the other hand, the Jiangsu Province is one of the most developed provinces, known for high industrial output. In 2007, the province had a serious water pollution incident [37]. With its recent rapid development, the province currently emits a huge amount of wastewater [38,39]. Considering this, the method we proposed allocated a lower emission permit to this province.

The proposed way of allocating emission permits could improve water quality, while providing equal development opportunities to the different regions. In addition, the proposed method can improve each province’s economic development potential as well as environmental quality. The proposed discharge permit allocation scheme should be complemented by building an effective emissions trading market to achieve even greater redistribution of total water pollution permits.

## 4. Conclusions

When allocating wastewater discharge permits, only considering each region’s current socioeconomic and environmental status is unfair because some regions have historically enjoyed low-cost pollution. This paper uses a new framework which considers each province’s economic, social, and environmental status together with emission history, to allocate China’s total COD discharge permits among its 30 provinces. 

The main conclusions extracted from the results of this study are the following. (1) Most western regions with poor economic status, such as Yunnan, Sichuan, and Guizhou, have small emission reductions, which means these provinces will be given higher discharge limits. (2) Industrial provinces, such as Jiangsu, Shandong, Guangdong, Liaoning, and Zhejiang, will be allocated high emission reduction rates. This is a way makes these provinces take responsibility for their high emissions in the past. (3) The economically developed provinces, such as Beijing, Tianjin, and Shanghai, should cut their emissions even further. Because these provinces have the higher economic capacity, making cuts can be affordable for them.

There is still much research needed in this area, for example, identifying a finer scale (province-basin level) for water pollution permit distribution that is relevant to each province’s water quality situation. In the future, our methodologies could also be applied to other wastewater discharges pollutants (for example N, P, BOD, or TOC).

Overall, we hope that this research provides valuable insights that can help the country’s policy-makers make sustainable, efficient, and fair emission reduction decisions.

## Figures and Tables

**Table 1:** The total wastewater discharge permits of each province based on the information entropy improved proportional allocation method.

Region	D_i_	D’_i_	q_i_ (×10^4^ Tons)	Reduction Rate (%)	Reduction Amount (×10^4^ Tons)	Target Emission Amount (×10^4^ Tons)
Beijing	0.584	1.307	16.200	13.580	2.200	14.000
Tianjin	0.505	1.130	20.900	11.744	2.454	18.446
Hebei	0.507	1.136	120.800	11.805	14.261	106.539
Shanxi	0.453	1.013	40.500	10.527	4.263	36.237
Inner Mongolia	0.437	0.978	83.600	10.166	8.499	75.101
Liaoning	0.533	1.194	116.700	12.403	14.474	102.226
Jilin	0.446	0.999	72.400	10.375	7.512	64.888
Heilongjiang	0.432	0.968	139.300	10.057	14.009	125.291
Shanghai	0.621	1.391	19.900	14.451	2.876	17.024
Jiangsu	0.666	1.492	105.500	15.497	16.349	89.151
Zhejiang	0.569	1.274	68.300	13.240	9.043	59.257
Anhui	0.376	0.841	87.100	8.742	7.614	79.486
Fujian	0.450	1.008	60.900	10.468	6.375	54.525
Jiangxi	0.327	0.732	71.600	7.610	5.449	66.151
Shandong	0.606	1.357	175.800	14.100	24.788	151.012
Henan	0.428	0.959	128.700	9.960	12.818	115.882
Hubei	0.419	0.938	98.600	9.742	9.605	88.995
Hunan	0.335	0.750	120.800	7.792	9.412	111.388
Guangdong	0.596	1.335	160.700	13.868	22.287	138.413
Guangxi	0.294	0.659	71.100	6.846	4.868	66.232
Hainan	0.428	0.959	18.800	9.963	1.873	16.927
Chongqing	0.423	0.948	38.000	9.849	3.743	34.257
Sichuan	0.240	0.537	118.600	5.575	6.613	111.987
Guizhou	0.337	0.754	31.800	7.837	2.492	29.308
Yunnan	0.287	0.642	51.000	6.667	3.400	47.600
Shaanxi	0.425	0.951	48.900	9.882	4.832	44.068
Gansu	0.417	0.933	36.600	9.697	3.549	33.051
Qinghai	0.402	0.900	10.400	9.355	0.973	9.427
Ningxia	0.452	1.012	21.100	10.513	2.218	18.882
Xinjiang	0.403	0.903	56.000	9.379	5.252	50.748
Sum	-	-	2210.6	-	234.101	1976.5

## Appendix A

**Table A1:** Province deviation coefficient calculation based on GDP.

Region	Historical Accumulated GDP Value (×100 Million Yuan)	Proportion of Historical Accumulated GDP Value	Historical Accumulated COD Emission (×10^4^ of Tons)	Proportion of History Accumulated COD Emission	GDP Deviation Coefficient
Beijing	68,487.332	0.035	227.680	0.009	0.247
Tianjin	33,307.772	0.017	259.640	0.010	0.580
Hebei	101,389.733	0.051	1355.520	0.051	0.994
Shanxi	40,971.367	0.021	613.620	0.023	1.114
Inner Mongolia	33,377.821	0.017	739.150	0.028	1.646
Liaoning	84,709.411	0.043	1290.640	0.049	1.133
Jilin	38,398.277	0.019	813.390	0.031	1.575
Heilongjiang	54,837.166	0.028	1279.090	0.048	1.734
Shanghai	85,952.625	0.044	436.710	0.016	0.378
Jiangsu	173,625.790	0.088	1489.130	0.056	0.638
Zhejiang	125,655.584	0.064	996.290	0.037	0.589
Anhui	57,896.228	0.029	926.530	0.035	1.190
Fujian	69,822.310	0.035	714.480	0.027	0.761
Jiangxi	42,382.114	0.021	847.040	0.032	1.486
Shandong	167,440.987	0.085	1787.050	0.067	0.794
Henan	105,801.436	0.054	1455.110	0.055	1.023
Hubei	75,082.773	0.038	1212.740	0.046	1.201
Hunan	73,901.622	0.037	1529.710	0.058	1.539
Guangdong	207,160.428	0.105	1947.250	0.073	0.699
Guangxi	43,694.758	0.022	1458.590	0.055	2.482
Hainan	10,464.425	0.005	194.460	0.007	1.382
Chongqing	35,612.424	0.018	477.880	0.018	0.998
Sichuan	77,807.276	0.039	1552.600	0.058	1.484
Guizhou	23,442.067	0.012	405.910	0.015	1.287
Yunnan	39,617.141	0.020	586.700	0.022	1.101
Shaanxi	41,425.898	0.021	626.320	0.024	1.124
Gansu	21,096.297	0.011	365.270	0.014	1.287
Qinghai	5978.322	0.003	114.670	0.004	1.426
Ningxia	7400.343	0.004	255.330	0.010	2.565
Xinjiang	29,119.675	0.015	616.820	0.023	1.575
Sum	1,975,859	1	26575.32	1	-

Note. Based on historical GDP and COD data and Equations (1)–(3), we calculated the GDP deviation coefficient.

**Table A2:** Population deviation coefficient calculation.

Region	History Accumulated Population Value (×10^4^ Person)	Proportion of History Accumulated Population Value	History Accumulated COD Emission (×10^4^ Tons)	Proportion of History Accumulated COD Emission	Population Deviation Coefficient
Beijing	28,055	0.013	227.68	0.009	0.641
Tianjin	19,287	0.009	259.64	0.010	1.063
Hebei	112,266	0.053	1355.52	0.051	0.954
Shanxi	55,143	0.026	613.62	0.023	0.879
Inner Mongolia	39,023	0.019	739.15	0.028	1.496
Liaoning	68,764	0.033	1290.64	0.049	1.482
Jilin	43,630	0.021	813.39	0.031	1.472
Heilongjiang	61,162	0.029	1279.09	0.048	1.652
Shanghai	33,146	0.016	436.71	0.016	1.041
Jiangsu	123,175	0.059	1489.13	0.056	0.955
Zhejiang	82,605	0.039	996.29	0.037	0.953
Anhui	97,540	0.046	926.53	0.035	0.750
Fujian	58,001	0.028	714.48	0.027	0.973
Jiangxi	70,029	0.033	847.04	0.032	0.955
Shandong	150,516	0.072	1787.05	0.067	0.938
Henan	151,616	0.072	1455.11	0.055	0.758
Hubei	91,624	0.044	1212.74	0.046	1.045
Hunan	104,973	0.050	1529.71	0.058	1.151
Guangdong	156,375	0.075	1947.25	0.073	0.984
Guangxi	76,132	0.036	1458.59	0.055	1.513
Hainan	13,586	0.006	194.46	0.007	1.130
Chongqing	45,935	0.022	477.88	0.018	0.822
Sichuan	133,330.9	0.064	1552.6	0.058	0.920
Guizhou	58,323	0.028	405.91	0.015	0.550
Yunnan	72,248	0.034	586.7	0.022	0.641
Shaanxi	59,417	0.028	626.32	0.024	0.833
Gansu	40,868	0.019	365.27	0.014	0.706
Qinghai	8849	0.004	114.67	0.004	1.023
Ningxia	9813	0.005	255.33	0.010	2.055
Xinjiang	33,521	0.016	616.82	0.023	1.453
Sum	2,098,953	1.000	26,575.32	1.000	-

**Table A3:** Water capital deviation coefficient calculation.

Region	History Accumulated Water Capital Value (×10^8^ Cubic Meter)	Proportion of History Accumulated Water Capital Value	History Accumulated COD Emission (×10^4^ Tons)	Proportion of History Accumulated COD Emission	Water Capital Deviation Coefficient
Beijing	378.32	0.001	227.68	0.009	8.215
Tianjin	199.32	0.001	259.64	0.010	17.782
Hebei	2260.62	0.006	1355.52	0.051	8.185
Shanxi	1560.51	0.004	613.62	0.023	5.368
Inner Mongolia	7264.67	0.020	739.15	0.028	1.389
Liaoning	4619.5	0.013	1290.64	0.049	3.814
Jilin	6372.08	0.018	813.39	0.031	1.743
Heilongjiang	12,246.1	0.034	1279.09	0.048	1.426
Shanghai	556.47	0.002	436.71	0.016	10.713
Jiangsu	6454.73	0.018	1489.13	0.056	3.149
Zhejiang	16,036.7	0.044	996.29	0.037	0.848
Anhui	11,475.66	0.032	926.53	0.035	1.102
Fujian	18,916.38	0.052	714.48	0.027	0.516
Jiangxi	24,678.45	0.068	847.04	0.032	0.469
Shandong	4583.72	0.013	1787.05	0.067	5.322
Henan	6263.97	0.017	1455.11	0.055	3.171
Hubei	14,928.7	0.041	1212.74	0.046	1.109
Hunan	27,604.61	0.076	1529.71	0.058	0.756
Guangdong	29,139.17	0.080	1947.25	0.073	0.912
Guangxi	30,383.02	0.084	1458.59	0.055	0.655
Hainan	5848.6	0.016	194.46	0.007	0.454
Chongqing	8812.11	0.024	477.88	0.018	0.740
Sichuan	39,638.64	0.109	1552.6	0.058	0.535
Guizhou	15,651.39	0.043	405.91	0.015	0.354
Yunnan	31,253.32	0.086	586.7	0.022	0.256
Shaanxi	6340.89	0.017	626.32	0.024	1.348
Gansu	3415.3	0.009	365.27	0.014	1.460
Qinghai	11,050.8	0.030	114.67	0.004	0.142
Ningxia	159.741	0.000	255.33	0.010	21.820
Xinjiang	14,683.12	0.040	616.82	0.023	0.573
Sum	362,776.6	1.000	26,575.32	1.000	-

**Table A4:** Each factor’s contribution to the allocation difference score in each province.

Region	Historical GDP Contribution (%)	Historical Population Contribution (%)	Historical Water Capital Contribution (%)
Beijing	29.3	41.3	29.5
Tianjin	14.8	51.0	34.2
Hebei	51.4	16.3	32.3
Shanxi	21.1	42.0	36.9
Inner Mongolia	17.1	50.4	32.5
Liaoning	40.4	30.8	28.8
Jilin	19.9	47.4	32.7
Heilongjiang	30.9	41.3	27.8
Shanghai	35.2	37.2	27.6
Jiangsu	68.8	9.4	21.8
Zhejiang	57.5	24.3	18.2
Anhui	37.8	29.4	32.8
Fujian	38.8	41.0	20.2
Jiangxi	30.4	49.5	20.0
Shandong	72.8	1.8	25.3
Henan	63.8	2.1	34.2
Hubei	45.1	29.0	25.9
Hunan	55.5	28.8	15.7
Guangdong	92.3	0.0	7.7
Guangxi	35.0	51.2	13.8
Hainan	2.9	62.6	34.6
Chongqing	19.1	49.0	31.9
Sichuan	82.0	18.0	0.0
Guizhou	14.2	54.6	31.2
Yunnan	32.1	55.1	12.8
Shaanxi	22.8	42.8	34.3
Gansu	9.9	52.0	38.1
Qinghai	0.0	68.9	31.1
Ningxia	0.9	60.9	38.3
Xinjiang	15.7	57.2	27.1

**Table A5:** COD discharge amounts in 2015 and 2020 and discharge reduction proportion.

Region	Discharge Amount (×10^4^ Tons) in 2015	2020 Based on Current Emission		2020 Consider Historical		2020 Country Plan	
		Emission Amount (×10^4^ Tons)	Cut Rate (%)	Emission Amount (×10^4^ Tons)	Cut Rate (%)	Emission Amount (×10^4^ Tons)	Cut Rate (%)
Beijing	16.2	14.1	12.8	14.0	13.6	13.9	14.4
Tianjin	20.9	18.4	12.0	18.4	11.7	17.9	14.4
Hebei	120.8	107.1	11.3	106.5	11.8	97.8	19
Shanxi	40.5	36.4	10.2	36.2	10.5	33.4	17.6
Inner Mongolia	83.6	74.7	10.6	75.1	10.2	77.7	7.1
Liaoning	116.7	102.2	12.4	102.2	12.4	101.1	13.4
Jilin	72.4	64.9	10.3	64.9	10.4	68.9	4.8
Heilongjiang	139.3	126.8	9.0	125.3	10.1	130.9	6
Shanghai	19.9	17.3	13.0	17.0	14.5	17.0	14.5
Jiangsu	105.5	87.5	17.1	89.2	15.5	91.3	13.5
Zhejiang	68.3	60.3	11.7	59.3	13.2	55.2	19.2
Anhui	87.1	79.4	8.9	79.5	8.7	78.5	9.9
Fujian	60.9	55.0	9.7	54.5	10.5	58.4	4.1
Jiangxi	71.6	67.1	6.3	66.2	7.6	68.5	4.3
Shandong	175.8	147.9	15.9	151.0	14.1	155.2	11.7
Henan	128.7	114.2	11.3	115.9	10.0	105.0	18.4
Hubei	98.6	88.7	10.1	89.0	9.7	88.8	9.9
Hunan	120.8	111.7	7.6	111.4	7.8	108.6	10.1
Guangdong	160.7	139.4	13.2	138.4	13.9	144.0	10.4
Guangxi	71.1	67.4	5.3	66.2	6.8	70.4	1
Hainan	18.8	17.0	9.7	16.9	10.0	18.6	1.2
Chongqing	38	34.1	10.2	34.3	9.8	35.2	7.4
Sichuan	118.6	111.0	6.4	112.0	5.6	103.4	12.8
Guizhou	31.8	29.4	7.6	29.3	7.8	29.1	8.5
Yunnan	51	48.0	6.0	47.6	6.7	43.8	14.1
Shaanxi	48.9	43.8	10.5	44.1	9.9	44.0	10
Gansu	36.6	33.1	9.5	33.1	9.7	33.6	8.2
Qinghai	10.4	9.5	8.7	9.4	9.4	10.3	1.1
Ningxia	21.1	19.0	10.1	18.9	10.5	20.8	1.2
Xinjiang	56	51.3	8.4	50.7	9.4	55.1	1.6
Sum	2210.6	1976.7	-	1976.5	-	1976.4	-

Note. Whole China COD emission in 2015 is 2213.5 × 10^4^ tons, The Discharge amount (×10^4^ tons) in 2015 here are not include Tibet (2.88 × 10^4^ tons) data. The cut rate (reduction rate) means the proportion of average COD amount one province need to cut in 2016–2020 compared with its 2015 COD emission.
